# Fullerenol protects cornea from ultraviolet B exposure

**DOI:** 10.1016/j.redox.2022.102360

**Published:** 2022-06-03

**Authors:** Xia Chen, Junling Yang, Minghui Li, Shuang Zhu, Maoru Zhao, Cao Yang, Bo Liu, Hui Gao, Ao Lu, Lingling Ge, Lingyue Mo, Zhanjun Gu, Haiwei Xu

**Affiliations:** a Southwest Eye Hospital, Southwest Hospital, Third Military Medical University (Army Medical University), Chongqing, 400038, China; b Key Lab of Visual Damage and Regeneration & Restoration of Chongqing, Southwest Eye Hospital, Southwest Hospital, Chongqing, 400038, China; c Clinical Medical Research Center, Southwest Hospital, Third Military Medical University (Army Medical University), Chongqing, 400038, China; d CAS Key Laboratory for Biomedical Effects of Nanomaterials and Nanosafety and CAS Center for Excellence in Nanoscience, Institute of High Energy Physics and National Center for Nanoscience and Technology, Chinese Academy of Sciences, Beijing, 100049, China; e College of Materials Science and Optoelectronic Technology, University of Chinese Academy of Sciences, Beijing, 100049, China

**Keywords:** Fullerenol, Ultraviolet B, Corneal protection, Free radicals

## Abstract

The eyes are highly susceptible to the oxidative stress induced by ultraviolet B (UVB, wavelength between 280 ∼ 320 nm), which could cause severe damage to the cornea. Fullerenols are effective antioxidants to alleviate UVB-induced injury, while their application for the eyes is still rare. In present study, we investigated the protective performance and mechanism of fullerenols on cornea under UVB radiation in vivo and in vitro. The synthesized fullerenols exhibited broad-spectrum free radical scavenging properties (applicable to both reactive oxygen species (ROS) and reactive nitrogen species (RNS)) and photo-stability. When compared with another widely used antioxidant glutathione (GSH), the administration of fullerenols markedly decreased the injured area, corneal edema, cell death, and increased the cell proliferation in UVB-induced rat cornea. The effects of fullerenols were confirmed in UVB-exposed human corneal epithelial cells (hCECs), where elevated cell viability and proliferation, decreased oxidative free radical production, repaired mitochondrial dysfunction and DNA lesions were observed. RNA sequencing (RNA-Seq) analysis demonstrated that fullerenol alleviated UVB-induced corneal injury through down-regulation of oxidative stress-related genes and up-regulation of proliferation-associated genes. Our results demonstrate the suitability of fullerenols as a potential exogenous treatment in ameliorating UVB-induced cornea damage.

## Highlights

• Fullerenol produces significant precaution on UVB-induced corneal damage.
• Fullerenol exhibits broader spectrum free radical scavenging properties than GSH.
• Fullerenol mitigates the LSCs loss in the cornea caused by UVB.
• Fullerenol down-regulates oxidative stress genes and up-regulates proliferation-related genes.

## Graphical abstract

**Figure:**
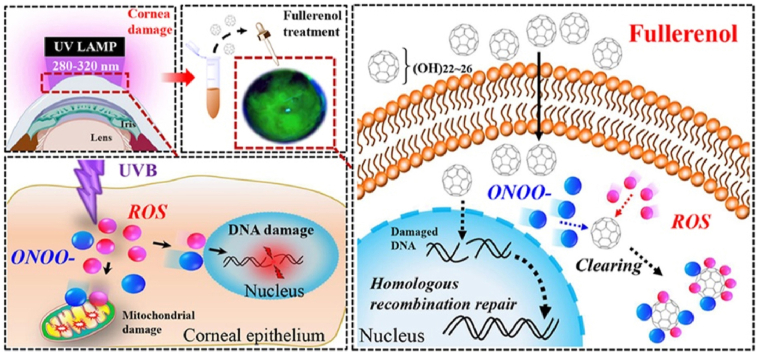

## 1 Introduction

The eye is the most sensitive organ in the human body to ultraviolet radiation aside from skin [1,2]. It is believed that the same ultraviolet rays from the sun, which can burn the skin, can also cause damage to ocular tissues, especially the cornea which is located at the anterior aspect of the eye [3,4]. Compared with ultraviolet A which can directly reach the retina, ultraviolet radiation at a shorter wavelength, designated as ultraviolet B (UVB, wavelength between 280 and 320 nm), is found to cause severe and long-term injury to the cornea, leading to complications such as opacification, edema, or photokeratitis [5,6]. One of the causes of ocular damage induced by UVB is the generation of reactive oxygen species (ROS)/reactive nitrogen species (RNS) and the decrease of corneal antioxidants [[7], [8], [9]]. Such a prooxidant/antioxidant imbalance may result in the oxidative injury of the cornea and further causes the damage to cellular lipids, proteins, or DNAs, and eventually leads to cell apoptosis [5,10]. Excessive free radicals also play a role in the induction of glaucoma by activating inflammatory pathways [8,11]. Therefore, considering the pathophysiological mechanism of UVB to the cornea, supplementation of an effective exogenous antioxidant that mitigates the oxidative stress might be essential to protect the cornea from UVB-induced damage.

Currently, there is no existent drug for UVB-induced cornea injuries in the clinic. Previously, antioxidant agents including enzymatic antioxidants (e.g. superoxide dismutase (SOD) or catalase) or small molecular antioxidants (e.g. glutathione (GSH) or vitamin E) were explored for the treatment of ocular disease [3,12]. However, based on their antioxidant mechanisms, most of these antioxidants have a limited spectrum for free radical scavenging [13,14]. For instance, SOD is particularly effective in scavenging superoxide anions (O_2_•^−^) [15,16] and vitamin E is only responsible for peroxyl radicals (LOO•) deprivation [17,18]. More importantly, long-term exposure to UVB is found to significantly decrease the activity of enzymatic antioxidants [12,19], which further hinders its application in protecting UVB-induced ocular damage. Therefore, a suitable UV protector with broad-spectrum, effective free radical scavenging ability and good chemical stability is desirable. Recently, fullerenol, also known as water-soluble fullerene derivatives, is extensively studied for direct free radical scavenging applications based on its large numbers of delocalized double *π* bonds with low energy unoccupied molecular orbitals [20,21]. It is called “radical sponges” owing to its effectiveness for not only scavenging a wide range of ROS including O_2_•^−^, hydroxyl (•OH), and lipid radicals particularly [22], but also inhibiting RNS by reacting directly with nitric oxide [20,23]. It is critical and unique, as some common antioxidants, represented by GSH, protect against lipid peroxidation and protein oxidation from injuries mediated only by ROS, but not RNS [24]. In addition to its broad-spectrum property, fullerenol scavenges free radicals more efficiently than conventional antioxidants such as vitamin E or SOD [25,26]. And apart from the activity mentioned above, fullerenol induces endogenous phase II antioxidant enzymes and modulates cell antioxidant status through upregulation of the Nrf/ARE-antioxidant pathway under oxidative stress stimulation [22]. Moreover, recent studies have shed light on the excellent peroxidase-like activities that fullerenol nanoparticles exhibit by the cycles of combining and dissociating with the peroxidase substrate in low pH environments [27]. Unlike other ROS scavengers, fullerenol also exhibits high stability in various physiological environments or under different kinds of irradiation stimuli.

**Scheme 1:**
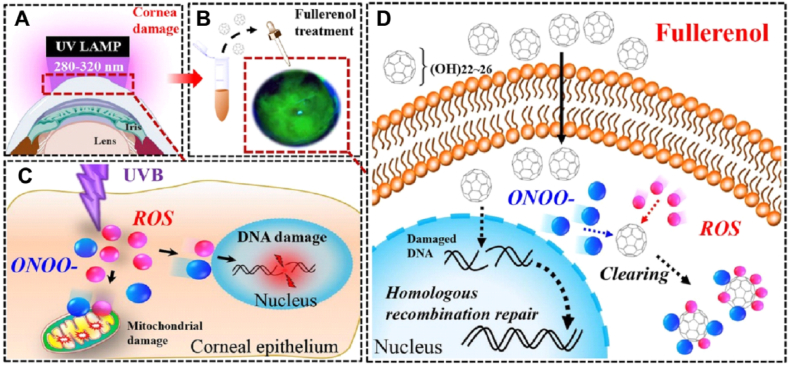
Graphical scheme of the experiments. The possible mechanism underlying UVB-induced corneal surface damages and the protection of fullerenol in corneal epithelial cell injury using in vitro and in vivo models. A, C) Schematic diagram of the corneal response to UVB radiation exposure. The corneal epithelial cells exposed to UVB produce reactive oxygen species (ROS) and peroxynitrite anion (ONOO^−^). The accumulation of free radicals is expected to cause oxidative stress and further single-strand breaks of DNA. Free radical-associated mitochondrial damage decreases mitochondrial membrane potential and disturbs the function of mitochondrial respiration chain. B, D) Schematic illustration of the protective mechanisms of fullerenol on the cell injury caused by UVB irradiation. Fullerenol easily penetrates the cell membrane and reacts with excessive radicals localized throughout the cytoplasm, quenches them by redox reactions. Upon oxidative DNA damage in nuclei, it traverses the nuclear envelope, promotes homologous recombination repair (HRR) and double-strand break repair.

As a broad-spectrum and effective free radical scavenger, fullerenol was reported to repress intracellular oxidative stress and rescue the HaCaT human skin keratinocytes from DNA damage when irradiated with UVB [28]. It also produced marked protection on radiation-injured gastrointestinal (GI) tract and skin [26,29]. By inhibiting oxidative stress and suppressing expression of key inflammatory cytokine TGF-β1, fullerenol was observed to block bleomycin-induced pulmonary fibrosis [30]. The powerful free radical scavenging capacity of fullerenol made it efficient in treating oxidative stress-related diseases such as Alzheimer's disease [31], intervertebral disk degeneration [32], and myocardial infarction [20]. In bone injury models, fullerenol was found to enhance the osteogenic differentiation of stem cells through activation of the FoxO 1/SOD2 pathway and expression of osteoclastogenesis genes, which promoted bone healing [33]. Through antagonizing TNF-α-induced ion channel activation and neuropeptide production, fullerenol was reported to relieve lumbar radiculopathy [34]. As oxidative stress is a key pathophysiological event of cornea injury induced by UVB exposure, it has not been clarified whether fullerenol will reconstruct the injured cornea through similar mechanisms. Therefore, we systematically investigated the protective performance and mechanism of fullerenol on cornea under UVB radiation in vivo and in vitro. The fullerenol was synthesized via a catalyst assistant mechanical chemistry. It exhibits broad-spectrum free radical scavenging properties (applicable to both ROS and RNS) and photo-stability. To illustrate the UVB-alleviating effect of fullerenol, GSH, a widely used antioxidant for ocular disease, was selected as a comparison [35]. In comparison with GSH, fullerenol exhibited a significantly enhanced therapeutic effect and reduced apoptosis level in UVB-radiation injured rat cornea and UVB-exposed human corneal epithelial cells (hCECs). Such therapeutic effect was largely based on its efficient antioxidant property that can decrease the production of free radicals and thus inhibit cellular mitochondria, DNA damage, and cell death. More interestingly, fullerenol was also found to increase the cell proliferation of both hCECs and limbal stem cells in the cornea of rats, which was considered to be a distinctive therapeutic function of fullerenol. RNA sequencing (RNA-Seq) was further conducted to gain a thorough understanding of the protective effect of fullerenol against hCECs to UVB exposure. As such, our work provides a comprehensive study to evaluate the suitability of fullerenol for protecting the cornea from UVB radiation, which may serve as a promising candidate for developing effective treatment of eyes for people, such as those who receive intense UVB exposure in high-altitude areas (see Scheme 1).

## 2 Experimental section

### 2.1 Synthesis and characterization of fullerenols

The fullerenols were synthesized according to a previous report by catalyst assistant mechanical chemistry strategy [26]. TEM (JEOL JEM2100plus) was applied to study the size and morphology of fullerenols. The hydrodynamic sizes were measured with a DLS particle size analyzer (Brookhaven Omni). The molecular structures of as-prepared fullerenols were characterized by Fourier transform infrared spectrometer (Thermo iN10-iZ10). The carbon-carbon double bonds on the carbon cage were studied by a high-resolution Raman spectrometer (HORIBA LabRAM HR Evolution).

### 2.2 1, 1-diphenyl-2-picrylhydrazyl (DPPH) radical scavenging test

A 100 μM DPPH radical working solution in ethanol was prepared. After mixing equal volumes of DPPH solution and fullerenols solution, the working concentrations of fullerenols were 6.25, 12.5, 25, and 50 μg/mL, respectively, and the mixture reacted in darkness for 30 min. UV–visual spectrophotometer (Hitachi) was applied to measure the absorption value of the mixture at 517 nm.

### 2.3 2,2'-azinobis (3-ethyl-benzothiaziline-6-sulfonate) (ABTS) radical scavenging test

ABTS radical solution was produced after 7 mM ABTS stock solution was reacted with 2.45 mM potassium persulfate in darkness at room temperature for 16 h and was diluted with PBS to obtain the ABTS working solution. After mixing equal volumes of ABTS and fullerenols solution, the working concentrations of fullerenols were 6.25, 12.5, 25, and 50 μg/mL, respectively, and the mixture reacted in darkness for 10 min. UV–visual spectrophotometer was applied to measure the absorption value of the mixture at 734 nm.

### 2.4 Peroxynitrite (ONOO^−^) scavenging test

ONOO^−^ solution was produced through the following steps. 5 mL of 1 M HCl was first added to 10 mL of 50 mM NaNO_2_ and 50 mM H_2_O_2_ in the ice bath, followed by 5 mL of 1.5 M NaOH, and yellow ONOO^−^ free radical solution was obtained after 10 min reaction, which was diluted 10 times to obtain the ONOO^−^ working solution. After mixing equal volumes of ONOO^−^ solution and fullerenols solution, the working concentrations of fullerenols were 6.25, 12.5, 25, and 50 μg/mL, respectively, and the mixture reacted in darkness for 2 min. UV–visual spectrophotometer was applied to measure the absorption value of the mixture at 302 nm.

### 2.5 •OH scavenging test

1 mM TMB solution with dimethyl sulfoxide (DMSO) as the solvent, 4 mM FeSO_4_ solution with HAc-NaAc buffer solution (pH 4.0) as the solvent, and 40 mM H_2_O_2_ aqueous solution were prepared. Then, equal volumes of TMB, H_2_O_2_, fullerenols, and FeSO_4_ were added in sequence and mixed, and the working concentrations of fullerenols were 12.5 and 25 μg/mL respectively. After 5 min in darkness, the absorption value of the mixture at 652 nm was measured by UV–visual spectrophotometry.

### 2.6 O_2_•^−^ scavenging test

1 mM nicotinamide adenine dinucleotide (NADH) aqueous solution, 0.25 mM NBT aqueous solution, and 15 μM (phenazine methosulfate) PMS aqueous solution were prepared. Then, 200 μL of PBS, 100 μL of NADH, 100 μL of NBT, and 100 μL of fullerenols were added in sequence and mixed, and the working concentrations of fullerenols were 25 and 50 μg/mL respectively. Finally, 100 μL PMS was added to activate the O_2_•^−^ formation reaction. UV–visual spectrophotometer was used to measure the change of the absorption value of the mixture solution at 560 nm within 10 min.

### 2.7 Animals

Sprague-Dawley (SD) rats, aged three weeks old, were obtained from the Third Military Medical University (Army Medical University) Animal Center. All animals were housed in a temperature-controlled room with a 12 h:12 h light: dark cycle with constant temperature (22–25 °C) and humidity (55 ± 5%). Animal preparation experimentation complied with all ethical regulations and each experiment used three individuals at least.

### 2.8 In vivo UVB radiation and antioxidant repair corneal radiation damage model test

The SD rats were divided into four groups: control group, UVB group, UVB + GSH (UG), and UVB + fullerenol (UF) treated group. The control group did not receive any irradiation and/or eye drop. In the other three groups of experiments, both eyes of each anesthetized rat were irradiated with a UVB lamp (Bioblock Scientific, Illkirch Cedex, France) at an average power of 612.1 μW/cm^2^ within 5 min of UV light exposure per day from a distance of 0.06 m. According to the product specification of the UVB lamp, the average peak wavelength is 280 nm, which is distributed within ±5 nm and in the UVB radiation spectrum. The rest of the eye surface was protected from UVB rays and the plane of the lamp was perpendicular to the optical axis of the eye. The rat corneal injury model based on single daily exposure to UVB was obtained after 5 consecutive days. GSH (6.8 μg/mL) and fullerenol (25 μg/mL) were added at the same molar concentration (22.16 μM) to the corneas of the rats in the last two groups respectively once a day after the fifth day of the modeling, which was marked as D0 (day 0). The rats were sacrificed at three observation time points: 5 (D0), 10 (D5), 14 (D9), and 21 (D16) days after the start of UVB exposure. Both irradiated and non-irradiated eyes were photographically documented and the corneas were employed for immunohistochemical or other examinations.

### 2.9 Corneal fluorescein staining

Corneal fluorescein staining was employed to detect whether the corneal epitheliums were defective. 1 h after the antioxidant restoration, the rats were anesthetized before 2 μL of 3% fluorescein were dropped into the conjunctival sac of each eye. The corneal epitheliums were examined and photographed 2 min later using a slit lamp under a Cobalt blue filter. The green area represents the corneal epithelial defect stained with fluorescein.

### 2.10 Anterior segment optical coherence tomography (AS-OCT)

AS-OCT (RTVue-100, Optovue, USA) was used to scan the cross-section of the cornea and capture corneal cross-section images. The eye tracker of the spectral domain OCT instrument was aimed at the center of the eyeball, then eight corneal cross-section images (0, 22.5, 45, 67.5, 90, 112.5, 135, and 157.5 axes; 6 mm scan-length) were acquired and analyzed. Five points separately selected from the bilateral limbus region of the cornea, mid and central zone of the cornea were recorded as 1, 2, 3, 4, and 5. The thickness of the corresponding points and especially the central corneal thickness (CCT) was calculated numerically.

### 2.11 TUNEL assay

The terminal deoxynucleotidyl transferase-mediated biotinylated UTP nick end labeling (TUNEL) assay (In Situ Cell Death Detection Kit, Roche, Basel, Switzerland) was used to detect the apoptosis of corneas. According to the manufacturer's protocol, a 50 μL TUNEL reaction mixture (buffers 1 and 2 were blended at a ratio of 1:9) was added to each frozen section of the corneas. After being incubated in a humidified atmosphere for 60 min at 37 °C in the dark, the slides were rinsed three times with PBS and incubated with DAPI. Finally, images were acquired by the confocal laser scanning microscope (Zeiss LSM 800 confocal microscope, ZEN Microsystems; ZEISS, Germany).

### 2.12 Cell culture

Human corneal epithelial cells (hCECs) were cultured by corneal limbal explant culture method and the human cornea was sourced from the Southwest Hospital Eye Bank, Third Military Medical University (Army Medical University) and were complied with ethics committee approval. Corneal tissue was peeled off from the human cornea, stripped into smaller pieces, and seeded into six-well plates pre-coated with human vitronectin (Gibco, USA). The explants were added to 40 μL fetal bovine serum (FBS; HyClone, China) before being placed in CO_2_ incubator overnight for further attachment. The cells culture medium consists of Dulbecco's Modified Eagle Medium: Nutrient Mixture F-12 (DMEM/F12; HyClone, China), 10% FBS (HyClone, China), and 1% penicillin-streptomycin (HyClone, China). The attached cells reached subconfluence when the density reached 70%–80%, then they were harvested with 0.05% Trypsin-EDTA (Gibco BRL, USA). The harvested cells were replated at a scale of 1:3 in T25 flasks and the culture medium was changed every three days.

### 2.13 Cell counting kit-8 (CCK-8) assay

The enhanced Cell Counting Kit-8 (Beyotime, China) to detect the viability of the hCECs in each group. In detail, the hCECs were cultured in 96-well plates at 37 °C in a humidified atmosphere containing 5% CO_2_ with DMEM/F12 High Glucose medium containing 10% FBS and 1% penicillin-streptomycin. After the density reached 70%–80%, the hCECs were irradiated by UVB radiation using the UV lamp set at a 2–3 cm vertical distance from the 96-well plates. The hCECs underwent radiation stress at different times and were washed with PBS and cultivation continued for 24 h with or without the added antioxidant agents GSH (22.16 μM) and fullerenol (22.16 μM). The cells in each group were incubated with 100 μl (per well) fresh medium consisting of a 1:10 ratio of CCK-8 solution and DMEM at 37 °C in 5% CO_2_ for 1 h. Optical density (OD) was recorded at 450 nm by the microplate reader (Varioskan Flash, Thermo Fisher, USA).

### 2.14 Immunofluorescence staining

Immunofluorescence was performed on both corneal cryosections and the hCECs. Individual corneal samples were collected on the 21st day after modeling (D16) when experiments were terminated. Immediately after, immunofluorescence staining of frozen tissue sections was performed as previously described [36]. Briefly, the eyeballs of the rats were enucleated and fixed in 4% paraformaldehyde (PFA; HyClone, China) for 30 min at 4 °C. After being taken out under a microscope (Olympus, Japan), the corneas were infiltrated with 30% sucrose and dehydrated overnight at 4 °C. Then the corneas were transferred to optimal cutting temperature solution (Sakura Finetek, USA) at −80 °C and cut into a 10-μm-thick sagittal slice by a freezing microtome (Thermo Fisher, Waltham, USA). After being rinsed three times with PBS and incubated in 0.3% Triton X-100 and 3% BSA for 30 min at 37 °C, the sections were incubated with the primary antibody, *anti*-caspase-3 antibody (1:400; Cell Signaling Technology, Danvers, MA, USA), *anti*-PH3 antibody (1:500; Abcam, Cambridge, UK)and CK15 (1:400; Cell Signaling Technology, Danvers, MA, USA), at 4 °C overnight, followed by incubation with the secondary antibody, Alexa Fluor 488 (1:500, Molecular Probes, Life Technologies, Eugene, OR, USA) or Alexa Fluor 568 (1:500, Thermo Fisher) for 2 h at 37 °C. Then the cell nuclei were stained with DAPI (Solarbio, Beijing, China).

The hCECs were seeded in 24-well culture plates plated with cell-climbing slices, fixed in 4% PFA for 30 min, and then washed three times with PBS. Fixed cells were incubated in 0.3% Triton X-100 and 3% BSA for 30 min before being incubated with *anti*-ki67 antibody (1:500; Abcam, Cambridge, UK), anti-8-OHdG antibody (1:200; Santa Cruz Biotechnology, America), *anti*-NRF2 antibody (1:1000; Cell Signaling Technology, USA), anti–HO–1 antibody (1:1000; Beyotime, China) and Phospho Histone H2A.X (Ser 139) (20E3) Rabbit mAb antibody γ-H2AX (1:400; Cell Signaling Technology, USA). The primary antibody was incubated overnight at 4 °C and the second antibody Alexa Fluor 568 was incubated for 2 h at 37 °C. Nuclei were also counterstained by DAPI and the slices were mounted with fluorescent anti-quenching reagents. Images of immunofluorescence staining were visualized and quantified under a confocal laser scanning microscope (Zeiss LSM 800 confocal microscope, ZEN Microsystems; ZEISS, Germany) and analyzed using Zeiss imaging software.

### 2.15 Intracellular oxidative stress assay

The ROS in the hCECs was labeled by cell-permeable fluorescent probes 2,7-Dichlorodihydrofluorescein diacetate (DCFH-DA) (Beyotime, China). The cells were cultured and treated, as described in the previous paragraph. Before being fixed and permeabilized, the hCECs were incubated with DCFH-DA at 5 μM for 30 min at 37 °C per the manufacturer protocol, followed by the stained of cell nuclei with DAPI for 10 min. The photographs were obtained by a confocal laser scanning microscope (Zeiss LSM 800 confocal microscope, ZEN Microsystems; ZEISS, Germany) and the ROS levels were measured via flow cytometry (BD FACS Calibur, San Jose, CA).

### 2.16 Intracellular peroxynitrite anion assay

BBoxiProbe® peroxynitrite anion detection kit (BestBio, China) was applied to the detection of peroxynitrite anions in the hCECs by fluorogenic probes BBoxiProbe® O71. The fluorescent dye was diluted 200 times by PBS and preheated at 37 °C before being added to the 24-well plate and incubated with the UV-treated cells. After 30 min the staining solution was removed and the hCECs were rinsed three times with PBS. The confocal laser scanning microscope (Zeiss LSM 800 confocal microscope, ZEN Microsystems; ZEISS, Germany) was used to monitor the distribution of green fluorescence, and photomicrographs were obtained.

### 2.17 Mitochondrial membrane potential assay

The mitochondrial membrane potential assay kit with JC-1 (JC-1; Beyotime, China) was employed to detect changes in the mitochondrial membrane potential (MMP). The hCECs were avoided light and incubated in JC-1 dyeing working fluid containing the JC-1 stock solution (200×) and assay buffer at a 1∶200 ratio. Then JC-1 dye was discarded and cells were washed three times with JC-1 buffer. Finally, the fluorescence images were acquired by the Zeiss confocal laser scanning microscope (Zeiss LSM 800 confocal microscope, ZEN Microsystems; ZEISS, Germany).

### 2.18 RNA-seq and bioinformatic analysis

The 12 samples which ensure the number of cells in each sample is 1 × 10^6^ and were divided into control group, UVB group, FU group, and GU group, with three samples of each respectively. TRIzolTM (Invitrogen, Carlsbad, CA, United States) was used for RNA extraction in the first pass and all mRNA was enriched by oligo (dT) magnetic beads, purified, and chemically fragmented by fragmentation buffer. Next, the high-quality RNA samples were used to construct transcriptome libraries. PCR amplification and the purification of the products were performed. Sequencing analysis of the target DNA fragment was on the Agilent 2100 platform. Relative gene expression levels were calculated as fragments per kilobase million mapped reads (FPKM). Changes in gene expression were identified and visualized using the R package DESeq2 (1.16.1). Statistically significant differentially expressed genes (DEGs) were defined and selected based on fold change >2 and adjusted p-value < 0.05. The Kyoto Encyclopedia of Genes and Genomes (KEGG) analysis was performed using the KEGG pathway database (https://www.genome.jp/kegg/pathway.html) to analyze the selected DEGs at the functional level.

### 2.19 Real-Time quantitative polymerase chain reaction (RT-qPCR)

The RT-qPCR tests were performed as described previously [37]. In brief, total RNA was extracted from 1 × 10^6^ hCECs with 1 mL of TRIzolTM (Sigma-Aldrich, St. Louis, MO, United States), 200 mL of chloroform, 500 mL of isopropanol and 1 mL of 75% ethyl alcohol. The concentration and purity of the RNA were detected using a spectrophotometric instrument (Thermo Fisher). Following the manufacturer's instructions, reverse transcription was performed using a Prime Script RT Reagent Kit (Takara, Tokyo, Japan) and qPCR was carried out with SYBR Green qPCR Mix (Takara Bio Inc, Japan) through a CFX96 Real-Time PCR System (Bio-Rad, Hercules, CA, United States). . The primers were produced by Sangon Biotech (Shanghai, China), and they are shown in Supplementary Material Table 1. The PCR conditions were as follows: 30 s at 95 °C, 41 cycles of 5 s at 95 °C, and 30 s at 60 °C followed by plate reading and then 10 s at 95 °C followed by a melting curve analysis (65–95 °C in increments of 0.5 °C per 5 s).

### 2.20 Western blotting

Western blotting analysis was performed following our previously described methods [38]. Protein levels of RAD51 (1:1000; Cell Signaling Technology, USA), NRF2 (1:1000; Cell Signaling Technology, USA), HO-1 (1:1000; Beyotime, China), α-Tubulin (1:1000, Beyotime) in hCECs that underwent different treatments were analyzed by Western. The cell lysates were subjected to sodium dodecyl sulfate (SDS) (Amresco, OH, USA)-PAGE and subsequently transferred to a polyvinylidene fluoride (PVDF) membrane (Millipore Immobilon, USA). Following washes with TBST, protein bands were detected using enhanced chemoluminescence ECL (Healthcare, Buckinghamshire, United Kingdom) according to the manufacturer's instructions. Relative protein expression levels were quantified using ImageJ software with α-Tubulin as control.

### 2.21 Statistical analysis

The statistical analyses were performed using GraphPad Prism 7.0 (GraphPad, CA, USA). Data obtained from different samples were presented as the Mean ± Standard Error of Mean (SEM). Statistical significance was evaluated by one-way ANOVA for multiple comparisons while Tukey's post-hoc tests were used for pairwise comparisons and defined as P < 0.05 (*), P < 0.01 (**), P < 0.001 (***), or P < 0.0001 (****).

## 3 Results

### 3.1 Characterization and evaluation of free radical scavenging performance of fullerenols

**Fig. 1:**
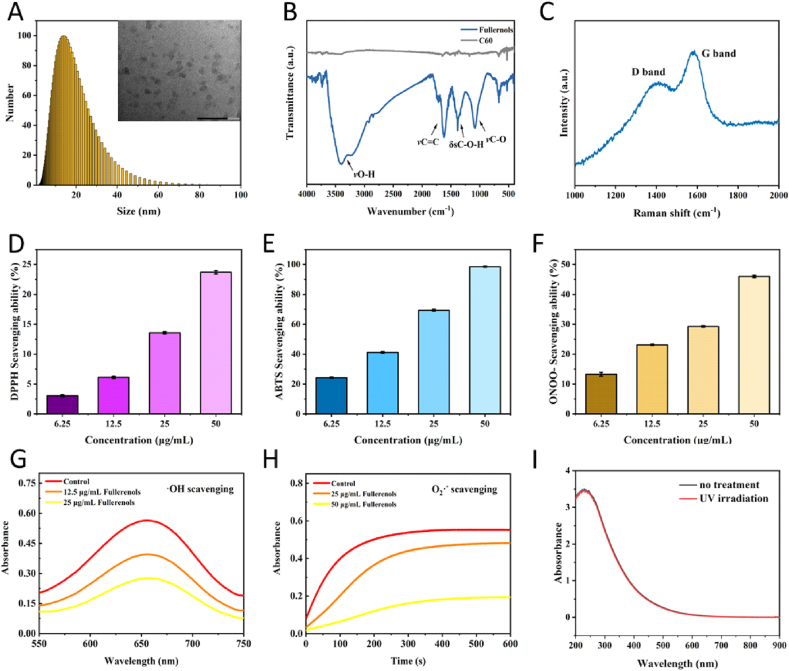
Characterization and evaluation of the free radical scavenging performance of fullerenols. A) hydrodynamic size of fullerenols (insert: TEM image, scale bar 100 nm). B) FTIR spectrum of fullerenols. C) Raman spectrum of fullerenols. D) DPPH radical scavenging ability of fullerenols. E) ABTS radical scavenging ability of fullerenols. F) ONOO^−^ scavenging ability of fullerenols. G) •OH scavenging ability of fullerenols. H) O2•^−^ scavenging ability of fullerenols. I) UV–vis absorption spectra of fullerenols treated with and without UV irradiation (15 W, 1 h).

Carbon nanomaterial fullerenols were synthesized by a facile synthetic method via catalyst assistant mechanical chemistry strategy according to our previous report [26]. According to the hydrodynamic size distribution, the diameter of as-prepared fullerenols was mainly 15 nm, which matched with the result of the transmission electron microscope (TEM) image (Fig. 1A). To confirm the molecular structure of fullerenols, the products were further characterized by Fourier transform infrared spectrometer (FTIR) and Raman spectrometer. As shown in Fig. 1B, compared to pure fullerene, the FTIR spectra of as-prepared fullerenols show four characteristic absorption peaks, including broad O–H stretching vibration (νO-H, 3450-3250 cm^−1^), C=C stretching vibration (νC = C, 1621 cm^−1^), O–H in-plane deformation vibration (δsC-OH, 1384 cm^−1^) and C–O stretching vibration (νC-O, 1083 cm^−1^). In the Raman spectrum, the two main peaks, 1360 cm^−1^ and 1595 cm^−1^, are consistent with the D and G bands of C=C on the carbon cage of fullerenols (Fig. 1C). The FTIR and Raman results confirmed the successful introduction of hydroxyl groups into carbon cages to form hydroxylated fullerenes. We thus studied the RNS/ROS suppressing ability of fullerenol to evaluate its suitability to serve as a UVB-protector for eyes. In terms of RNS, fullerenols had a strong scavenging ability on model nitrogen radical DPPH, ABTS, as well as ONOO^−^ in a concentration-dependent manner (Fig. 1D–F, the scavenging rates of these three free radicals can reach 23.71%, 98.55%, and 45.99%, respectively at 50 μg/mL of fullerenols). Moreover, fullerenols also showed excellent ROS scavenging performance. It could effectively clean up the superoxide anions (O_2_•^−^) generated by nicotinamide adenine dinucleotide (NADH)/phenazine methosulfate (PMS) reaction and block the conversion of p-nitro-blue tetrazolium chloride (NBT) into blue formazan, which reduced the absorbance at 560 nm (Fig. 1G). Additionally, fullerenols could also block the hydroxyl radicals (•OH) produced by Fenton's reaction and inhibit the production of oxidized 3,3″,5,5′-tetramethylbenzidine (oxTMB, dark green), thereby reducing the absorbance at 652 nm (Fig. 1H). We also studied the photostability of fullerenols under UV irradiation. As shown in Fig. 1I, UV–visible light absorption of fullerenols confirmed its stability even under high intensity and long duration conditions (15W, 1 h) of UV irradiation, which further indicated its stability in protecting UV-induced injury. It showed that 25 μg/mL fullerenol scavenged •OH、O_2_•^−^and even ONOO^−^.

### 3.2 Therapeutic effect of fullerenol on the corneal injury of rats caused by UVB radiation

**Fig. 2:**
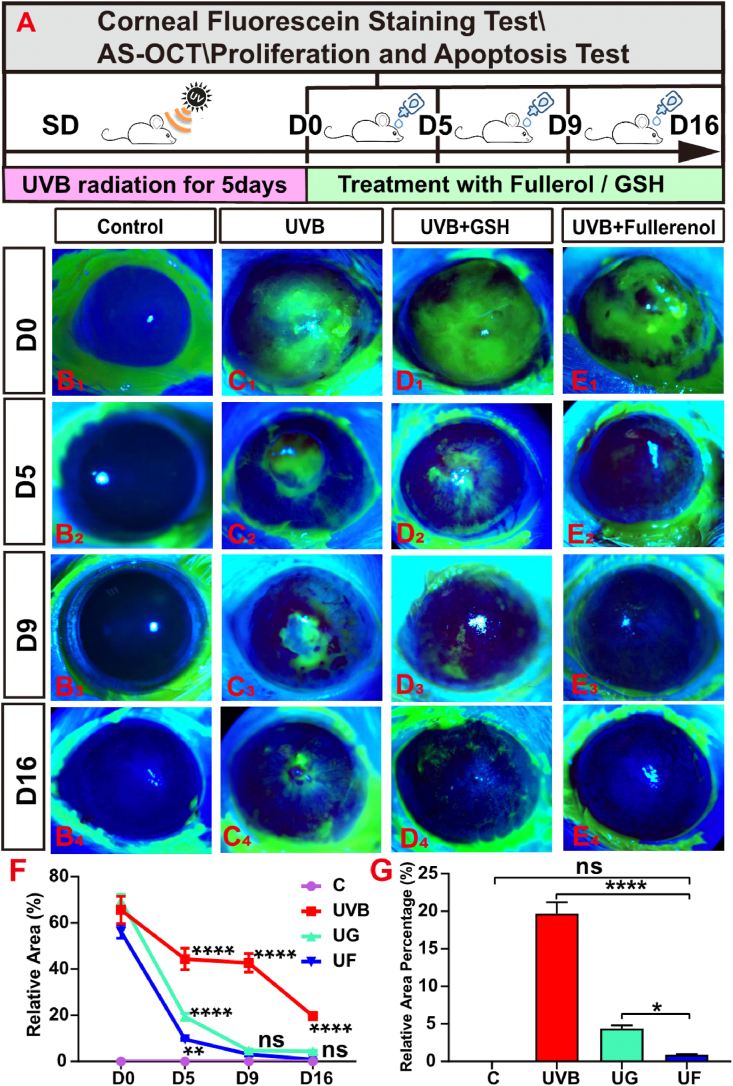
Therapeutic effect of fullerenol or GSH on the injuried area of corneal damage caused by the UVB radiation in rats. A) Schematic diagram of UVB radiation-induced corneal injury and treatment with 25 μg/mL fullerenol and 6.8 μg/mL GSH (the same molar concentration). The rat cornea was irradiated with UVB for 5 days to establish the model. B_1_-E_4_) The representative images of corneal damages under cobalt blue light of control, UVB, GSH + UVB, fullerenol + UVB group investigated by fluorescein sodium staining respectively. B_1_–B_4_) The control group was observed on days 0, 5, 9 and 16 respectively after treatment. C_1_–C_4_) The UVB group. D_1_-D_4_) The UVB + GSH group. E_1_-E_4_) The UVB + fullerenol group, all were observed on days 0, 5, 9 and 16 respectively after treatment. F) Quantitative comparison of relative damaged area of central corneal in different groups. G) Comparison of the injured area between fullerenol and GSH treatment for 16 days (n = 3 eyes/group). The above detections were implemented in three independent experiments. Data were expressed as the mean ± SEM from three independent experiments. **P < 0.01, ****P < 0.0001 using one-way ANOVA and post-hoc Tukey's test. (For interpretation of the references to colour in this figure legend, the reader is referred to the Web version of this article.)

We thus evaluated the protective effect of fullerenol on UVB-induced cornea damage on Sprague-Dawley (SD) rats via fluorescein sodium staining, anterior segment optical coherence tomography (AS-OCT), apoptosis, and the proliferation test (Fig. 2A). As shown in Fig. 2C, when the eyes were exposed to 5 consecutive days of UVB radiation, a distinct green area that represented corneal epithelial defect under cobalt blue light was observed in the central regions of the corneas. Although the cornea could repair the injury (green area decreased over time) by itself, there was still a significant difference in damage area between the UVB group (Fig. 2C and 19.67 ± 1.25% damage area) and the control group (Fig. 2B and 0.88 ± 0.08% damage area) on day 16. In contrary, UVB + GSH (UG, 69.65 ± 2.18% damage area)) and UVB + fullerenol (UF, 56.19 ± 2.33% damage area) treated group showed considerable curative effect starting from D5 (Fig. 2C), while fullerenol treatment showed a significantly faster and more complete therapeutic effect than GSH treatment (Fig. 2E). Compared to the UG group which still presented 4.36 ± 0.37% of relative cornea damage at the end of the experiment, the UF group exhibited almost no noticeable damage at D16 (no statistical significance between control and UF at D16, damage area 0.88 ± 0.08%) (Fig. 2F and G).

**Fig. 3:**
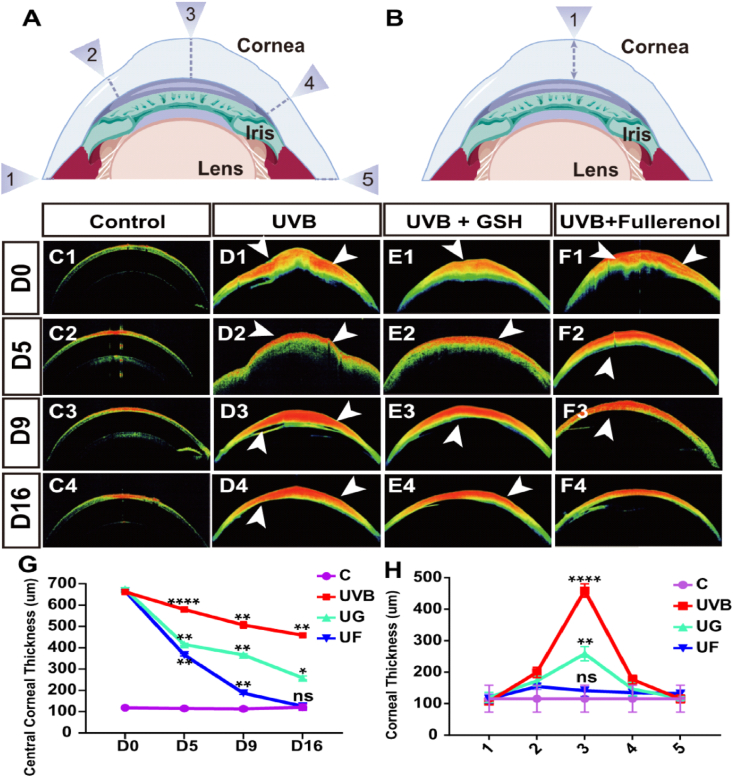
Effects of fullerenol or GSH on the corneal edema of UVB radiation-induced corneal injury in rats. A) Schematic diagram of the measurement of corneal thickness in the rats. B) Measurement of central corneal thickness. C–F) Anterior segment OCT images of control, UVB, GSH + UVB, fullerenol + UVB group, respectively (the white arrow indicated corneal edema). C_1_–C_4_) Control group; D_1_-D_4_) UVB group; E_1_-E_4_) UVB + GSH group; F_1_–F_4_) UVB + fullerenol group; G) Comparison of thickness in central corneal epithelium treated with fullerenol and GSH at different time points after UVB exposure (n = 3 eyes/group). H) Statistical analysis of the thickness of the whole cornea at 16 day after treatment with fullerenol or GSH (n = 3 eyes/group). The above detections were implemented in three independent experiments. Data were expressed as the mean ± SEM from three independent experiments. **P < 0.01, ***P < 0.0001, ****P < 0.0001 using one-way ANOVA and post-hoc Tukey's test.

Corneal epithelial thickness (CET) is another important parameter to evaluate corneal diseases [36], which can be measured with AS-OCT imaging (Fig. 3A and B). Numbered arrows are used in Fig. 3A and B to represent the sites where the corneal thickness was measured. In general, the corneas would allow higher transmission for the unscattered incidence light (Fig. 3C). However, the phenomenon of obvious corneal epithelial defect disrupting corneal structure as well as enhancing CET and edema (white arrow) was observed after UVB radiation (Fig. 3D). These abnormalities could be further reversed with the treatment of antioxidants GSH or fullerenol (Fig. 3E and F). As shown in Fig. 3G, fullerenol treatment realized almost complete normalization of the corneal thickness and disappearance of corneal edema (Fig. 3F), while obvious corneal edema was still observed after 16 days of GSH treatment, especially in the central cornea (Fig. 3E). The final central corneal thickness difference between the UG group (258.30 ± 7.38 μm) and UF (125 ± 10.80 μm) group was also statistically significant (Fig. 3H).

Taken together, as evidenced by the fluorescein staining test and AS-OCT result, both fullerenol and GSH showed a curative effect on damaged rat corneas from exposure to UVB, but fullerenol showed a much better therapeutic effect than that of GSH.

### 3.3 Effects of fullerenol on the apoptosis in the cornea of UVB radiation-induced rats

**Fig. 4:**
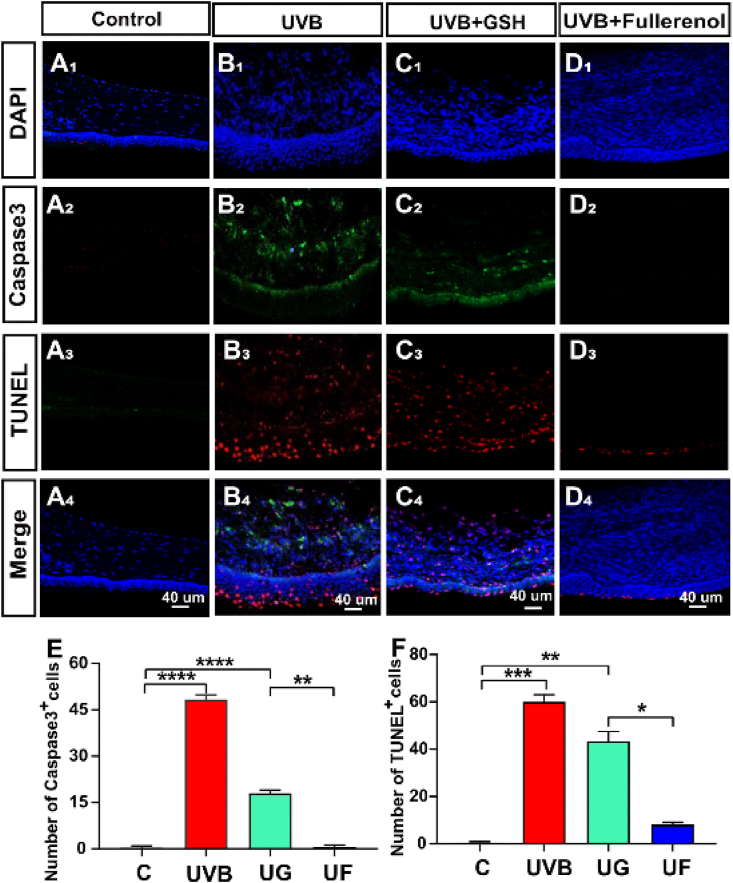
Effects of fullerenol or GSH on the apoptosis in the cornea of UVB radiation-induced rats. A-D) Apoptosis detection with immuno-fluorescence staining: A_1_-A_4_) Control group. B_1_–B_4_) UVB group. C_1_–C_4_) UVB + GSH group. D_1_-D_4_) UVB + Fullerenol group. E) Quantitative analysis of the number of Caspase3-positive cells in the cornea of rats. F) Quantitative analysis of the number of TUNEL-positive cells in the cornea of rats. The above detections were implemented in three independent experiments. Data were expressed as mean ± SEM from three independent experiments. *P < 0.1, **P < 0.01, ***P < 0.001, ****P < 0.0001 using one-way ANOVA and post-hoc Tukey's test. Effects of fullerenol on the apoptosis in the cornea of UVB radiation-induced rats.

Subsequently, we studied the apoptosis of keratocyte in rats, where the apoptotic markers of TUNEL and caspase3 were measured. Caspase3^+^ and TUNEL^+^ cells were rarely observed in the corneas of the control group (Fig. 4A), while a large number of caspase3^+^ and TUNEL^+^ cells were found in the UVB group (Fig. 4B), indicating that UVB radiation-induced apoptosis in the corneas. When it came to therapeutic effects, fullerenol treatment showed considerable alleviation of UVB-induced corneal apoptosis, where the number of caspase3^+^ cells in the UF group decreased markedly (Fig. 4E and 0.67 ± 0.47, P < 0.0001), and the number of TUNEL^+^ cells in the fullerenol-treated rats was much less than that in the GSH-treated group (Fig. 4F and 8 ± 0.81 vs 43.34 ± 3.40, P < 0.01). These results suggested that the fullerenol could reduce cell apoptosis, thereby mitigating corneal damage. Apart from the antiapoptotic effect, we also measured the influence of fullerenol on cell proliferation.

### 3.4 Effects of fullerenol on the cell proliferation in the different areas of cornea of UVB radiation-induced rats

**Fig. 5:**
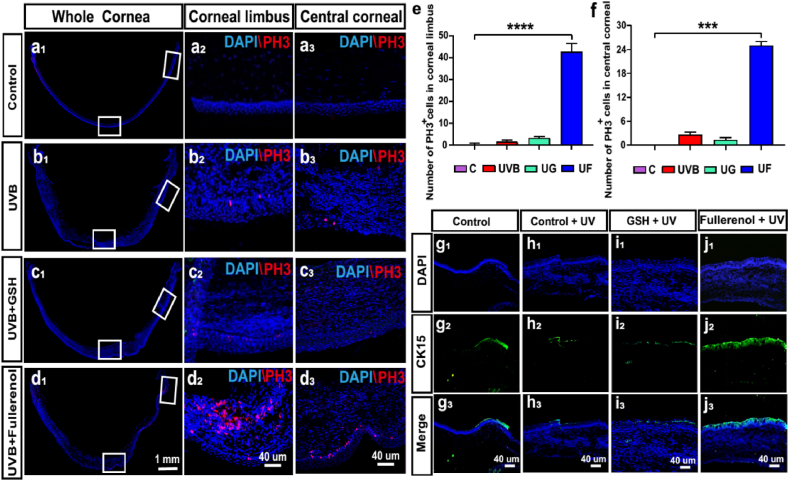
Effects of fullerenol or GSH on the cell proliferation of corneal epithelial cells and limbal stem cells in the cornea of UVB radiation-induced rats. a-d) Representative images of immunofluorescence for PH3 (red) and DAPI (blue) in different groups: a_1_-a_3_) Control group. b_1_–b_3_) UVB group. c_1_–c_3_) UVB + GSH group. d_1_-d_3_) UVB + Fullerenol group. a_1_, b_1_, c_1_, d_1_) Representative images of immunofluorescence for whole cornea. a_2_, b_2_, c_2_, d_2_) Representative images of immunofluorescence for cornea limbus. a_3_, b_3_, c_3_, d_3_) Representative images of immunofluorescence for central cornea. e) Comparison of the number of PH3 -positive cells in the cornea limbus. f) Comparison of the number of PH3 -positive cells in the central cornea. N = 3 samples per group. g-j) Limbal stem cell marker CK15 detection with immuno-fluorescence staining: g_1_-g_3_) Control group. h_1_–h_3_) UVB group. i_1_–i_3_) UVB + GSH group. j_1_-j_3_) UVB + Fullerenol group. Data were expressed as mean ± SEM from three independent experiments. *P < 0.1, ***P < 0.001, ****P < 0.0001 using one-way ANOVA and post-hoc Tukey's test. (For interpretation of the references to colour in this figure legend, the reader is referred to the Web version of this article.)

Both limbal and central cornea were marked by the white boxes on each image (Fig. 5a_1_-d_1_) and the corresponding magnified images were displayed in Fig. 5a_2_-d_2_ and a_3_-d_3_. It showed that neglectable PH3^+^ (mitosis marker) cells were observed in control (Fig. 5a), UVB (Fig. 5b) groups, while fullerenol treatment significantly increased the number of PH3^+^ cells in both limbal and central cornea (Fig. 5d,e and f). Moreover, the effect was not observed in GSH treated (Fig. 5c).

As presented in Fig. 5g, healthy limbus possessed a certain amount of CK15 positive limbal stem cells (LSCs) (Fig. 5g). After UVB exposure, the number of CK15 positive cells was significantly reduced (Fig. 5h). However, fullerenol significantly improved the number of CK15 positive cells (especially at limbal, Fig. 5j). It suggested that fullerenol may not only be able to mitigate the LSC loss in the basal layer of the cornea caused by UVB, but also effectively activate the stem cell characteristics and the endogenous proliferation of limbal stem cells. However, this effect on cell proliferation was not observed in GSH treated group (Fig. 5i).

Collectively, the protective mechanism of fullerenol to cornea upon UVB irradiation on rats, probably via both inhibiting the apoptosis pathway and activating the proliferation of stem cell.

### 3.5 Influences of fullerenol on the cell viabilities and proliferation of hCECs exposed to UVB

**Fig. 6:**
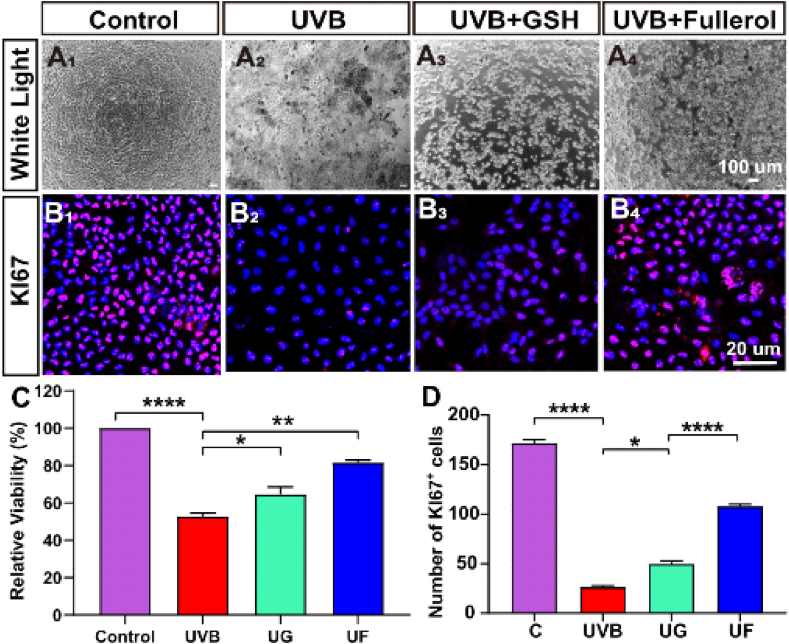
Influences of fullerenol or GSH on the cell viabilities and proliferation of hCECs exposed to UVB. A_1_-A_4_) Morphological features of hCECs under phase-contrast microscopy, showing morphological changes following 2h exposure to UVB and treated with 25 μg/ml fullerenol and 6.8 μg/ml GSH (the same molar concentration) for 24 h. B_1_–B_4_) Representative images of immunofluorescence for Ki67 (red) and DAPI (blue) in different groups: B_1_) Control group. B_2_) hCECs exposed to UVB for 2 h. B_3_) hCECs treated with GSH for 24 h. B_4_) hCECs treated with fullerenol for 24 h. C) Cell counting kit (CCK-8) detected cell viability of hCECs treated with fullerenol and GSH for 24 h respectively after UVB radiation damage G) Comparison of the number of Ki67-positive cells in different groups. The above detections were implemented in three independent experiments. Data were expressed as mean ± SEM from three independent experiments. *P < 0.1, **P < 0.01, ****P < 0.0001 using one-way ANOVA and post-hoc Tukey's test. (For interpretation of the references to colour in this figure legend, the reader is referred to the Web version of this article.)

We further confirmed the protection of fullerenol on primary isolated hCECs [36,39]. We firstly studied the safety of fullerenol to hCECs, and found that the cell activity was not influenced even with a dose as high as 400 μg/mL after 24h (Fig. S2A). In our study, 25 μg/mL fullerenol was selected for the subsequent experiments. In the cellular experiment, the UVB apparatus and experimental setup are shown in Figs. S2B–C and hCECs were treated with fullerenol and GSH (the same molar concentration as fullerenol) after UVB radiation. As presented in Fig. 2, Fig. 6h UVB radiation-induced severe cytotoxicity, while fullerenol greatly increased cell viability level to over 80%. Both GSH and fullerenol could reverse this toxicity induced by UVB while fullerenol again exhibited much more normal spindle-shaped cells (Fig. 6A). Confocal images and quantification of the Ki67 (proliferation marker) staining are shown in Fig. 6B. As hCECs possess a high proliferative activity while rat cornea lacks such property. In contrast to the control group, a marked reduction was found in the UVB group, suggesting that UVB could greatly suppress cell proliferation. However, fullerenol was effective in enhancing cell proliferation, as the number of Ki67 positive cells (108 ± 1.63) was significantly higher than those of UVB (26.34 ± 1.25) and the UG group (49.67 ± 2.63) (Fig. 6B, D). It showed that fullerenol repaired UVB-induced cell death and increase cell proliferation.

### 3.6 Changes of ROS and ONOO^−^ level in the hCECs exposed to UVB and the protective effect of fullerenol

**Fig. 7:**
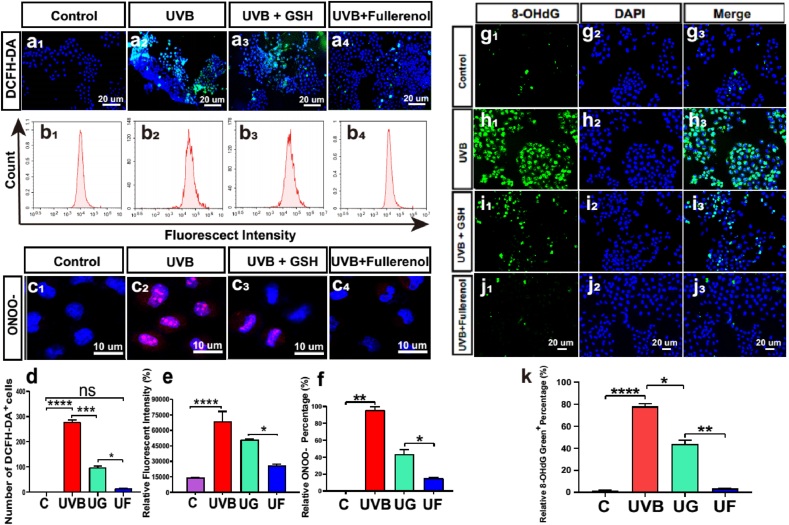
Changes of ROS and ONOO^−^ level in the hCECs exposed to UVB and the effect of fullerenol or GSH. a_1_-a_4_) Representative images of immunofluorescence for intracellular ROS (DCFH-DA, green, counterstained with DAPI, blue) in hCECs. a_1_) Control group. a_2_) The UVB group. a_3_) hCECs treated with 6.8 μg/ml GSH after exposure to UV for 2 h a_4_) hCECs treated with 25 μg/ml fullerenol after exposure to UV for 2 h b_1_-b_4_). Flow cytometry analysis of the intracellular ROS production treated with fullerenol and GSH after UVB radiation damage. b_1_) Control group. b_2_) The UVB group. b_3_) UVB + GSH group. b_4_) UVB + fullerenol group. c_1_-c_4_) Representative images of hCECs fluoresced in yellow (ONOO^−^) and blue (DAPI). c_1_) Control group. c_2_) hCECs exposed to UV for 2 h c_3_) hCECs treated with GSH for 24h. c_4_) hCECs treated with fullerenol for 24 h. d) Comparison of the number of ROS-positive cells in different groups. e) The relative fluorescence intensity of ROS production in each group was analyzed with flow cytometry. f) Comparison of the number of ONOO^−^ positive cells. g-k) Representative images of immunofluorescence for 8-OHdG (green, counterstained with DAPI, blue): g_1_-g_3_) Control group. h_1_-h_3_) UVB group. i_1_-i_3_) UVB + GSH group. j_1_-j_3_) UVB + Fullerenol group. k) Quantitative analysis of the number of 8-OHdG-positive cells. The above detections were implemented in three independent experiments. Data were expressed as the mean ± SEM from three independent experiments. N = 3 samples per group. *P < 0.1, **P < 0.01, ****P < 0.0001 using one-way ANOVA and post-hoc Tukey's test. (For interpretation of the references to colour in this figure legend, the reader is referred to the Web version of this article.)

As radiation damage usually results in oxidative stress response [40,41]. The fullerenol neutralized ROS/RNS efficiently based on a large number of delocalized double π [26,42]. We thus evaluated the level of ROS and RNS after different treatments. DCFH-DA probe was used for ROS assessment. As shown in Fig. 7a and d, relative fluorescent intensities in UVB-irradiated hCECs were markedly increased compared to the control group, reflecting the dramatical intracellular ROS production after UVB exposure. The fluorescence signals of UG and UF groups were decreased, while the reduction of fluorescent cells was more significant in UF treatment, which was nearly comparable to the control group. In addition, flow cytometry was utilized to confirm the effect on ROS, which was shown to be in line with each other (Fig. 7b and e). Furthermore, we assessed RNS production by detecting the levels of ONOO^−^ in the hCECs. As displayed in Fig. 7c and f, a large amount of ONOO^−^ production was observed after UVB exposure, posing potential oxidative injury and apoptosis of the irradiated hCECs. Although both GSH and fullerenol could reduce the ONOO^−^ level, fullerenol treatment (relative fluorescence expression 15 ± 1.13%) displayed much stronger ONOO^−^ inhibition than GSH treatment (relative fluorescence expression 44 ± 4.32%). Together with the outcomes of ROS, fullerenol exhibited enhanced intracellular free radical scavenging ability (both ROS and RNS) than GSH.

Oxidative damage to DNA was assessed by 8-hydroxydeoxyguanosine (8-OHdG) immunofluorescence staining. As shown in Fig. 7g-k, the number of 8-OHdG positive cells was increased after UVB irradiation, achieving more than 80 per field of view, while the cells in the control group only showed a few faint spots of staining (Fig. 7g). The 24 h GSH-treated group displayed a visible reduction in the number of 8-OHdG positive cells. The 8-OHdG positive cells were almost completely disappeared after fullerenol treatment, which demonstrated complete normalization of the level of total oxidized DNA. Hence, these results suggested that fullerenol restored the repair capacity of hCECs through suppression of UVB-induced oxidative DNA damage.

### 3.7 Influences of fullerenol on the DNA oxidative damage and antioxidant capacity of hCECs exposed to UVB

**Fig. 8:**
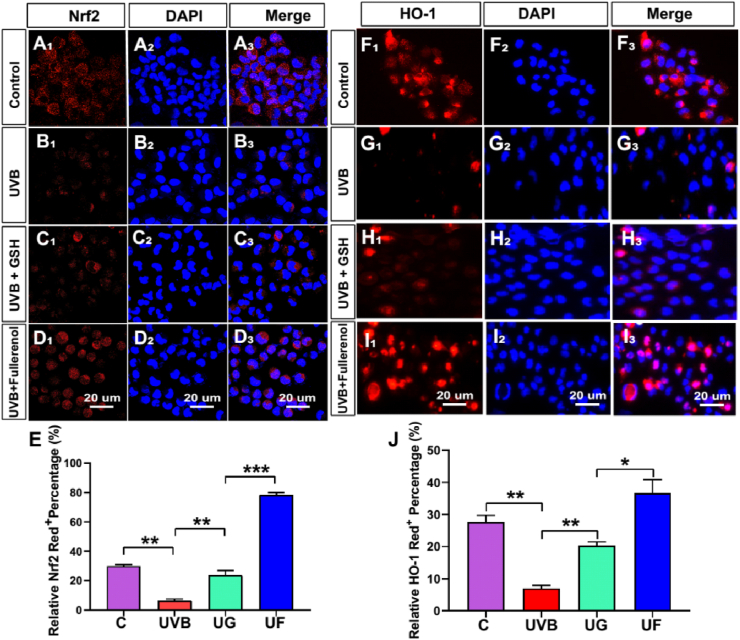
Influences of fullerenol or GSH on the DNA oxidative damage and antioxidant capacity of hCECs exposed to UVB. A-D) Representative images of immunofluorescence for Nrf2 (red) and DAPI (blue) in different groups: A_1_-A_3_) Control group. B_1_–B_3_) UVB group. C_1_–C_3_) UVB + GSH group. D_1_-D_3_) UVB + Fullerenol group. E) Quantitative analysis of the number of Nrf2-positive cells of different groups. F–I) Detection of antioxidant protein HO-1 with immuno-fluorescence staining following exposure to UVB and the effect of GSH or fullerenol: F_1_–F_3_) Control group. G_1_-G_3_) UVB group. H_1_–H_3_) UVB + GSH group. I_1_–I_3_) UVB + Fullerenol group. J) Quantitative analysis of the number of HO-1-positive cells in different groups. Data were expressed as the mean ± SEM from three independent experiments. *P < 0.1, **P < 0.01, ***P < 0.001, ****P < 0.0001 using one-way ANOVA and post-hoc Tukey's test. (For interpretation of the references to colour in this figure legend, the reader is referred to the Web version of this article.)

It showed that Nrf2 (red) was distributed both in the cytoplasm and in the nucleus of hCECs in the control group (Fig. 8A_1_-A_3_). Irradiated with UVB, the ratio of Nrf2 positive cells decreased significantly (Fig. 8B_1_-B_3_, P < 0.01), while both GSH and fullerenol treatment markedly blocked the reduction of Nrf2 positive cells ratio and there were significant differences between these two groups (Fig. 8C_1_-C_3_, Fig. 8D_1_-D_3_, Fig. 8E, P < 0.01). Compared to the UG group, it showed that increased nuclear distribution ratio of Nrf2 in the UF group (Fig. 8D_1_-D_3_). HO-1 is a downstream factor regulated by Nrf2 [43], it demonstrated that UVB irradiation markedly decreased the ratio of HO-1 positive cells, which was reversed by fullerenol treatment (Fig. 8F_1_-I_3_, J). These results were further confirmed by WB analysis (Figs. S5C–F). It suggested that fullerenol activated the Nrf2/HO-1 pathway to modulate UVB-induced oxidative stress and produced cytoprotective effects on the hCECs.

### 3.8 Fullerenol repaired DNA damage and mitochondrial membrane potential changes of hCECs caused by UVB radiation

**Fig. 9:**
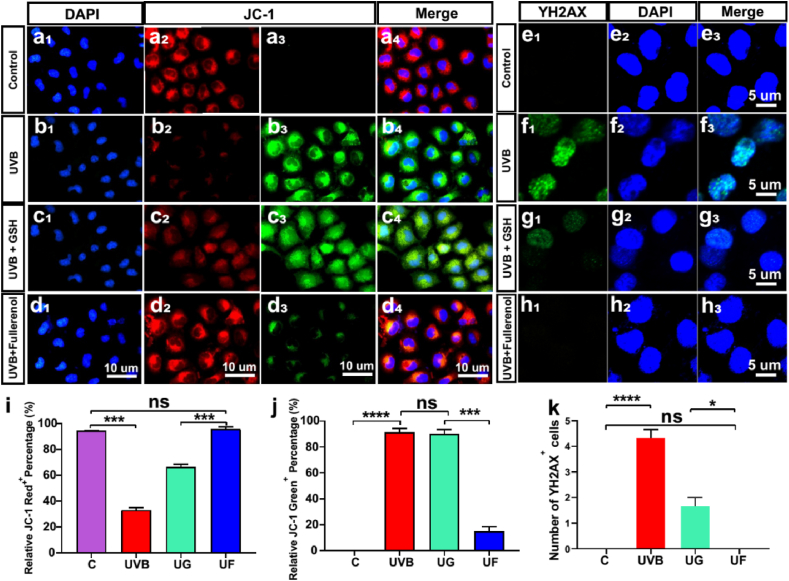
Effects of fullerenol or GSH on mitochondrial membrane potential changes and DNA damage in hCECs caused by UVB exposure. a-d) Fluorescence changes of different mitochondrial membrane potentials in cells after different exposures. Normal mitochondria were fluorescently labeled in red and the fluorescence changes from green to red when the mitochondrial membrane potential decreases. a_1_-a_3_) Control group. b_1_-b_3_) hCECs exposed to UV for 2 h. c_1_–c_3_) hCECs treated with 6.8 μg/ml GSH after exposure to UV for 2 h. d_1_-d_3_) hCECs were treated with 25 μg/ml fullerenol for 24 h. e-h) Representative images of immunofluorescence for the γ-H2AX positive cells (green, counterstained with DAPI, blue) in hCECs. e_1_-e_3_) Control group.f_1_–f_3_) hCECs exposed to UV for 2 h. g_1_-g_3_) hCECs treated with GSH for 24 h. h_1_–h_3_) hCECs were treated with fullerenol for 24 h after being exposed to UV radiation for 2 h. i) Comparison of the number of JC-1^+^ red cells in different groups. j) Comparison of the number of JC-1^+^ Green cells in different groups. k) Comparison of the number of γ-H2AX-positive cells in different groups. N = 3 samples per group. Data were expressed as the mean ± SEM from three independent experiments. *P < 0.1, ***P < 0.001, ****P < 0.0001 using one-way ANOVA and post-hoc Tukey's test. (For interpretation of the references to colour in this figure legend, the reader is referred to the Web version of this article.)

As free radicals directly attack mitochondria to activate the mitochondrial pathway of apoptosis or cause double-stranded double-stranded DNA to influence DNA replication or transcription, ultimately leading to cell apoptosis and death [44,45]. We then evaluated the mitochondrial membrane potential (MMP) which can reflect the mitochondrial membrane integrity as well as mitochondrial function [46,47]. When hCECs were exposed to UVB radiation, a dramatic decrease in red fluorescence of JC-1 and an increase of green fluorescence in the cytosol were detected, indicating a significant drop in MMP (Fig. 9b). In contrast, fellerenol treatment realized a significant enhancement in red fluorescence, which was even comparable to the control group (Fig. 9a, d and i). In terms of green fluorescent, only fullerenol greatly reduced its level while the quantified green fluorescence intensity of the GSH group remained as high as the UVB group (Fig. 9c, d and j). The variations in JC-1 expression levels indicated that fullerenol had a strong ability in reversing UVB-induced mitochondria damage.

When it came to DNA damage assessment, γ-H2AX staining assay was employed to determine the double-stranded DNA breaks (DSBs) [48]. As shown in Fig. 9f, UVB-induced severe DNA damage in hCECs, was evidenced by the highest green fluorescence of γ-H2AX. After treatment of fullerenol, the fluorescence intensity decreased sharply (comparable to the control group) (Fig. 9e, H and K), indicating that fullerenol could effectively repair DNA damage in hCECs from UVB radiation. Taken together with the above observations, our results demonstrated that fullerenol could suppress the production of toxic free radicals and repair the mitochondria and DNA damage in hCECs, which were associated with UVB radiation. And it further inhibited apoptosis pathways and finally promoted cell survival.

### 3.9 Screening of critical molecular pathways involved in protection of fullerenol on the UVB exposed hCECs

**Fig. 10:**
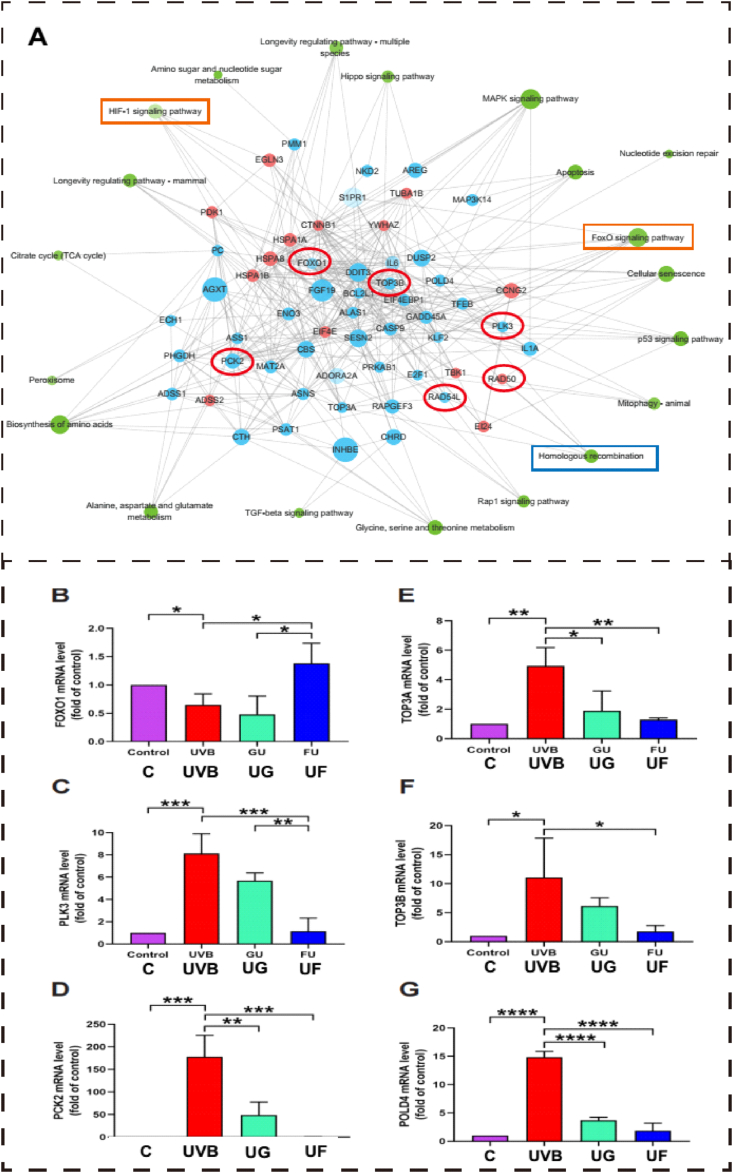
Kyoto Encyclopedia of Genes and Genomes (KEGG) analysis of molecular pathways involved in the protection of fullerenol on the UVB exposed hCECs. A) KEGG pathway analysis for differently expressed mRNAs. Each node of the green circle represents a signaling pathway. Grey linear represents interactive relationships between two signaling pathways. Each node of the blue circle represents down-regulated genes and the red circle represents up-regulated genes. The red and oval boxes represent the pathways and genes that differ the most in this group. B-D) The relative mRNA expression of genes associated with the FoxO signaling pathway (B–D) or homologous recombination pathway (E-G) which were involved in the protection of fullerenol in the UVB irradiated hCECs screened by RNA-Seq. B) FOXO1 mRNA expression. C) PLK3 mRNA expression. D) PCK2 mRNA expression. E) TOP3A mRNA expression. F) TOP3B mRNA expression. G) POLD4 mRNA expression. N = 3 samples per group. Data were expressed as the mean ± SEM from three independent experiments. *P < 0.1, ****P < 0.0001 using one-way ANOVA and post-hoc Tukey's test. (For interpretation of the references to colour in this figure legend, the reader is referred to the Web version of this article.)

To clarify the underlying molecular mechanism and provide evidence to confirm the protective effect of fullerenol, RNA-seq was conducted to screen the critical molecular pathways. The distribution of differentially expressed genes (DEGs) and cluster heat map analysis of each treatment group are presented in Fig. S3. The expression profile for all the genes was plotted as a clustering heatmap and shown in Fig. S3A, where the expression level of the homeologs in triple samples exhibited a consistent trend. Venn diagrams (Fig. S3B) revealed 8660 shared DEGs between different treatments and the volcano plots (Fig. S3C) showed the changes in transcriptome by pairwise comparison. The volcano plot showed the variation of up-regulated genes in different groups. To better illustrate the molecular mechanism of UF treatment, we further identified the top 20 significantly altered signaling pathways between UF and UVB groups (Fig. S4). The most significantly changed biological processes were analyzed by constructing a pathway interaction network map based on the Kyoto Encyclopedia of Genes and Genomes (KEGG) pathway analysis (Fig. 10A). As indicated in Fig. 10, DEGs were closely associated with apoptosis (e.g. apoptosis/autophagy/cellular senescence signaling), oxidative stress (MAPK signaling, TGF-β Signaling Pathway), DNA repairment pathways (homologous recombination, DNA replication), and cellular proliferation (e.g. cell cycle/p53 signaling).

To investigate whether fullerenol regulated antioxidant defense and homologous recombination repair play a critical role in protection against UVB irradiation in hCECs, we analyzed the transcriptional levels of target genes using RT-PCR. It showed that the mRNA levels of FOXO1 decreased significantly 24 h after UVB irradiation, fullerenol treatment reversed the decline of FOXO1 expression levels while GSH failed (Fig. 10B). However, the expression of PLK3 and PCK2 were significantly increased after UVB irradiation, which was inhibited after 24 h of GSH treatment or fullerenol treatment (Fig. 10C and D). The mRNA levels of TOP3A, a member of the 1A subfamily of DNA topoisomerases gene [49], were increased markedly in UVB-irradiated hCECs (Fig. 10E), and the levels of TOP3B and POLD4 were substantially blocked by fullerenol treatment (Fig. 10B–F). Western blot was used to verify homologous recombination repair (HRR)-related proteins and oxidative stress-related proteins. RAD51 is recruited to the damaged DNA in the cells [50] and is involved in the DNA repair [51]. It showed that UVB-irradiation markedly decreased the level of RAD51, while both fullerenol and GSH blocked the decline of RAD51 in UVB-irradiated hECEs(Fig. S5).

## 4 Discussion

The present study indicated that synthetic fullerenol showed its broad free radical scavenging ability. We demonstrated the superior ability of fullerenol in alleviating corneal damage induced by UVB radiation, which may result from inhibiting the apoptosis pathway, increasing cell proliferation, and activating limbal stem cell expression. Fullerenols have a delocalized *π*-conjugated structure, which endows fullerenols with efficient and broad-spectrum free radical scavenging ability. Previously, it has been reported that the generation of RNS and ROS is one of the main causes of UVB-induced ocular injury [52,53]. ONOO^−^ is a short-lived RNS produced intracellularly by the diffusion-controlled reaction of nitric oxide (NO^−^) with superoxide (O_2_•^−^) [54,55]. The ONOO^−^ level is closely related to various serious diseases and UVB-induced cornea injury [56,57]. It was confirmed in the present study when we utilized primary hCECs to analyze the outcome obtained from in vivo level. It showed that fullerenol suppressed the toxic free radical (ROS/RNS) production and repaired the mitochondria and DNA damage that were associated with UVB radiation in hCECs, which further inhibited apoptosis pathways and finally promoted cell survival. A previous study showed that fullerenol activated the Nrf2/HO-1 signaling pathway and increased the antioxidative capacity in the cardiomyocytes of rats during acute myocardial ischemia-reperfusion injury [58]. It confirmed in the present study that fullerenol activated the Nrf2/HO-1 pathway to relieve UVB-induced oxidative stress, which contributed to its cytoprotective effects on hCECs.Comprehensively, fullerenol with high antioxidant property protected the corneal from the damage caused by UVB radiation. This is consistent with the previous result, in which the skin radioprotective performance of 25 μg/mL fullerenol was demonstrated by both in vitro and in vivo experiments [26]. The experimental apparatus is sketched in Fig. S1. The 25 μg/mL fullerenol was chosen in the present study and the 6.8 μg/mL GSH was selected to serve as a control at the same molar concentration. In the cornea, limbal stem cells (LSCs) are the source of corneal epithelium regeneration [59], while UV radiation usually causes the loss and dysfunction of the LSCs, resulting in limbal stem cell deficiency (LSCD) [60]. Nevertheless, continuous LSCs renewal contributes to wound healing of corneal epithelium as well as maintains the homeostasis of the intraepithelial corneal epithelium. Therefore, we then investigated the influence of fullerenol on LSCs using the specific marker CK15 on rat cornea [61].

It is verified that elevated oxidative stress could lead to cell apoptosis by causing damage to mitochondria and DNA breaks [36,62,63]. In our study, a cationic fluorescent dye JC-1 was used to examine the loss of MMP. Under normal circumstances, most mitochondria display red fluorescence by JC-1 aggregates, and a typical green fluorescence of J-monomers would appear when mitochondria become depolarized [64,65].

The molecular mechanism of the protective and pro-proliferation ability of fullerenol was further illustrated by RNA-Seq analysis, where we found the down-regulation of cellular oxidative stress levels and up-regulation of cell proliferation-related genes play a major role. Interestingly, the homologous recombination pathway which related to DNA repair was screened as the candidate pathway involved in the fullerenol protection in UVB-induced injury in hCECs. Previous studies have shown that homologous recombination entails accurate resolution of double-strand breaks in endogenous DNA damages resulting from ROS-mediated oxidation and other metabolic processes [[66], [67], [68]]. It demonstrated in the present study that fullerenol completely reversed DNA damage in hCECs induced by UVB irradiation. In addition, apart from oxidative stress-related pathways, the expression of cell proliferation-associated pathways such as cell cycle, Rap 1, or oocyte meiosis signaling pathways were also considerably altered in fullerenol treated groups compared with UVB groups. Collectively, consistent with our preceding results, these data provided genetic evidence to support that fullerenol repairs the hCECs damage exposed to UVB irradiation by decreasing cellular oxidative stress levels and increasing cell proliferation, and thus realizes decreased apoptosis and cell death.

Considering its rigid chemical stability can free its practical application from adding a preservative, our work provides new insights into novel strategies for the treatment of LSCD and is a promising candidate for developing commercial eye drops to protect people who receive over-exposure to UVB radiation.

## Author contributions

X.C. and J.Y. contributed equally to this work. Xia Chen, Junling Yang: Conceptualization; Data curation; Formal analysis; Investigation; Methodology; Writing - Original Draft. Minghui Li, Shuang Zhu, Maoru Zhao, Cao Yang, Bo Liu, Hui Gao, Ao Lu, Lingling Ge: Formal analysis; Investigation; Methodology; Software; Visualization. Lingyue Mo: Editing of English grammar and syntax of the manuscript. Zhanjun Gu: Supervision; Writing - review & editing. Haiwei Xu: Project administration; Resources; Funding acquisition; Supervision; Writing - review & editing.

## Declaration of competing interest

The authors declare that they have no known competing financial interests or personal relationships that could have appeared to influence the work reported in this paper.

## Appendix A Supplementary data

UV irradiation instrument and influences of fullerenol on the cell viabilities. Distribution of DEGs and Cluster heat map analysis of fulleneol and GSH repaired hCECs after UVB radiation. (PDF).
