# Supplementary material for: Fullerenol protects cornea from ultraviolet B exposure
Source: Redox Biol. 2022 Jun 3;54:102360. doi: 10.1016/j.redox.2022.102360 (PMC9190064; doi:10.1016/j.redox.2022.102360)
Supplement: Multimedia component 1 [file mmc1.docx]

**Supporting Information**

**Fullerenol protects eyes from Ultraviolet B exposure**

Xia Chen^a,b,c,1^ , Junling Yang^a,b,1^, Minghui Li^a,b^, Shuang Zhu^d,e^, Maoru Zhao^d,e^, Cao Yang^a,b^, Bo Liu^a,b^, Hui Gao^a,b^, Ao Lu^a,b^, Lingling Ge^a,b^, Lingyue Mo^a,b^ Zhanjun Gu^d,e, **^,and Haiwei Xu^a,b,^ ^*^

^a^Southwest Eye Hospital, Southwest Hospital, Third Military Medical University (Army Medical University), Chongqing, 400038, China

^b^Key Lab of Visual Damage and Regeneration & Restoration of Chongqing, Southwest Eye Hospital, Southwest Hospital, Chongqing 400038, China

^c^Clinical Medical Research Center, Southwest Hospital, Third Military Medical University (Army Medical University),Chongqing 400038, China

^d^CAS Key Laboratory for Biomedical Effects of Nanomaterials and Nanosafety and CAS Center for Excellence in Nanoscience, Institute of High Energy Physics and National Center for Nanoscience and Technology, Chinese Academy of Sciences, Beijing, 100049, China

^e^College of Materials Science and Optoelectronic Technology, University of Chinese Academy of Sciences, Beijing, 100049, China

^*^Corresponding Author at: Southwest Eye Hospital, Southwest Hospital, Third Military Medical University (Army Medical University), Chongqing, 400038, China

^**^Corresponding Author at: CAS Key Laboratory for Biomedical Effects of Nanomaterials and Nanosafety and CAS Center for Excellence in Nanoscience, Institute of High Energy Physics and National Center for Nanoscience and Technology, Chinese Academy of Sciences, Beijing, 100049, China

E-mail addresses: haiweixu2001@163.com (H.Xu), zjgu@ihep.ac.cn(Z.Gu).

^1^These authors contributed equally to this work

Competing Interests: The authors declare that they have no known competing financial interests or personal relationships that could have appeared to influence the work reported in this paper.

**This PDF file includes:**

Supplemental **Fig. S1 to S6**


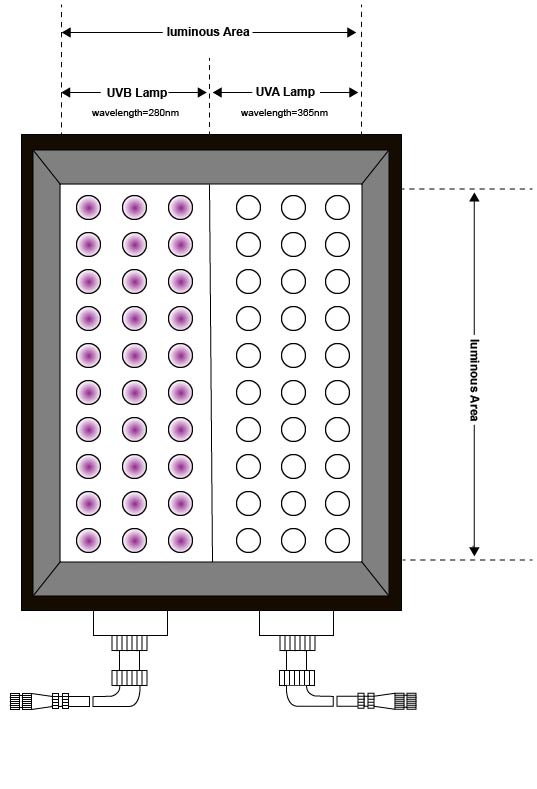


**Fig. S1.** Graphical scheme of UVB irradiation apparatus. The light emitting area of the irradiation apparatus is a rectangle shape area contained two distinct chambers. The UVB irradiation chamber contained a bank of 30 UVB lamps that emit wavelengths between 265 and 285 nm, with peak emission at 280 nm, according to the manufacturer.


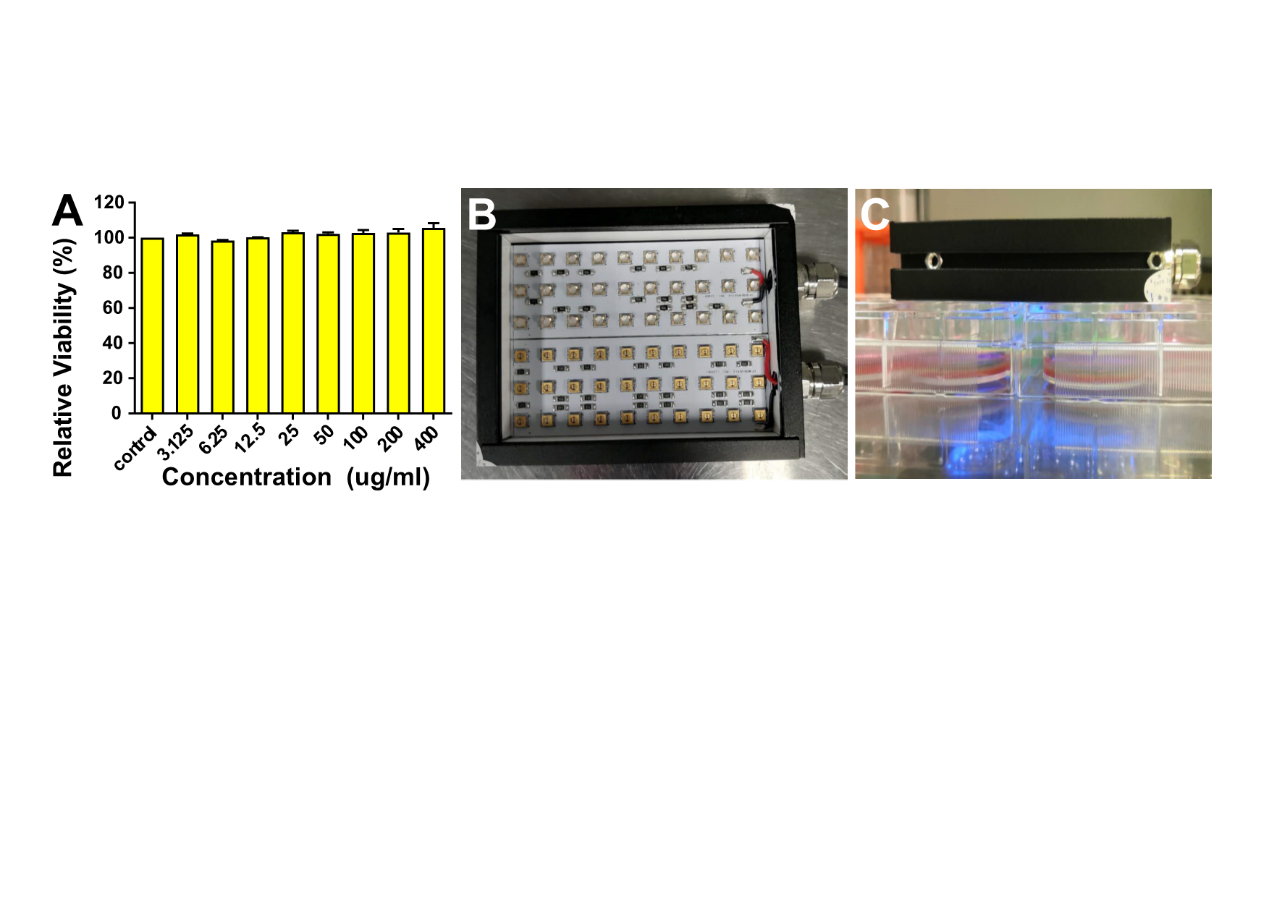


**Fig. S2.** UV irradiation instrument and influences of fullerenol on the cell viabilities. A) Cell counting kit (CCK-8) deteced cell viabilities of hCECs incubated with fullerenol for 24 h. B) Physical picture of UV irradiation instrument. The hCECs were irradiated with UVB. C) The above detections were implemented in three independent experiments. Data are the mean ± SEM from three independent experiments. *P < 0.1, **P<0.01, ****P < 0.0001 using one-way ANOVA and post-hoc Tukey’s test.

**Table 1 List of Primers**

| **Name** | **Forward primer** | **Reverse primer** |
| --- | --- | --- |
| **GAPDH** | CCATGTTCGTCATGGGTGTGA | CATGAGTCCTTCCACGATACCA |
| **FOXO1** | GGATGTGCATTCTATGGTGTACC | TTTCGGGATTGCTTATCTCAGAC |
| **PLK3** | TTTTCGCACCACTTTGAGGAC | GAGGCCAGAAAGGATCTGCC |
| **PCK2** | AGTAGAGAGCAAGACGGTGAT | TGCTGAATGGAAGCACATACAT |
| **TOP3A** | TGTAAGGCTGTAAAGCCCAATC | GATCAGGCTCGGTCAGGTTT |
| **TOP3B** | TGGCGAGAAGACCGTGTTC | AATCACCGTATTTCCCCTGGA |
| **POLD4** | ATCACTGATTCCTACCCGGTT | AGAGATGCCAGAGACTGCACT |

**Table 1** Table 1 List of Primers

**
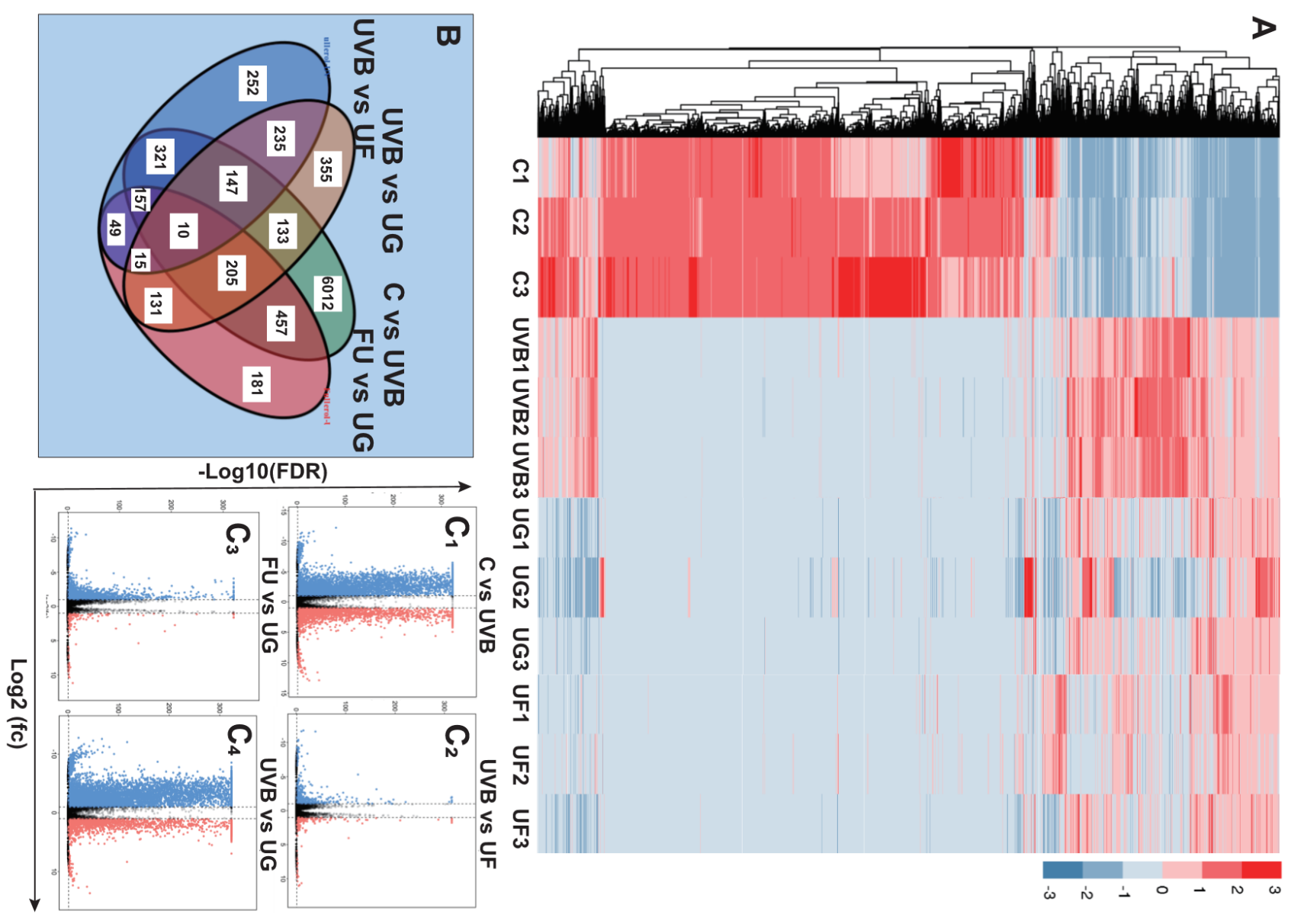
**

**Fig. S3.** Distribution of DEGs and Cluster heat map analysis of fulleneol and GSH repaired hCECs after UVB radiation. A) The differentially expressed mRNAs in the C group, the UVB group, the FU group, the GU group were classified and clustered. Each row represents a single mRNA and each column represents one tissue sample. Red, high relative expression; blue, low relative expression. P-values and Q-values (MA-plot) represent the significance of the differences (fold change2 and adjusted P-value0.05). B) The Venn map of the DEGs of each group. C_1_-C_4_) Volcano plot of all DEGs the groups compared to each other, C_1_) the C group compared with the UVB group; C_2_) the UVB group compared with the FU group; C_3_) the FU group compared with the GU group; C_4_) the UVB group compared with the GU group. Red: indicated up-regulated genes; Blue: indicated down-regulated genes. The closer the genes were to each other, the greater the difference.


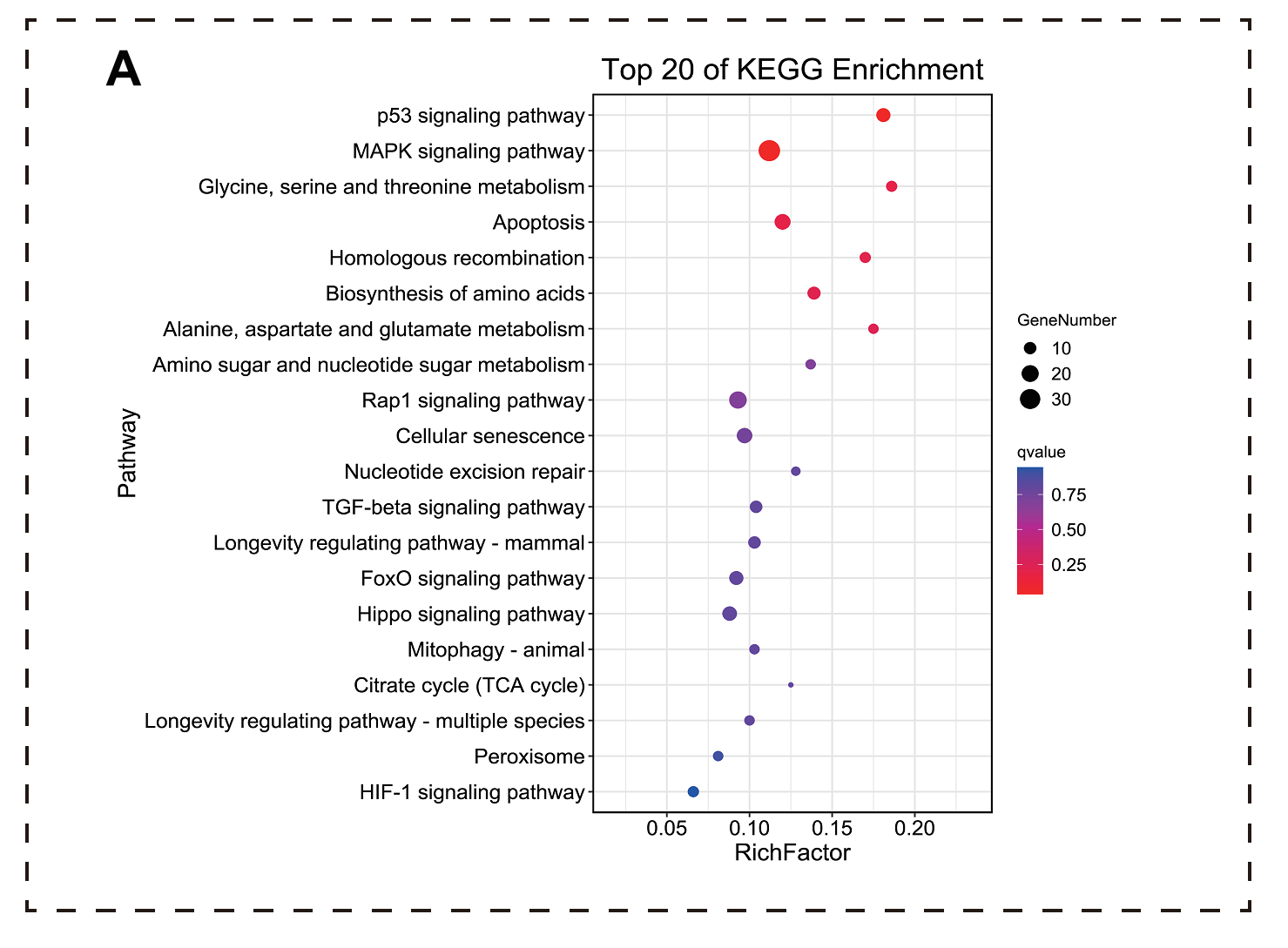


**Fig. S4.** The 20 most significantly altered pathways in the Kyoto Encyclopedia of Genes and Genomes (KEGG). The 20 most significantly changed biological processes in the KEGG enrichment analyses of UVB group compared with the UVB + Fullerenol group.


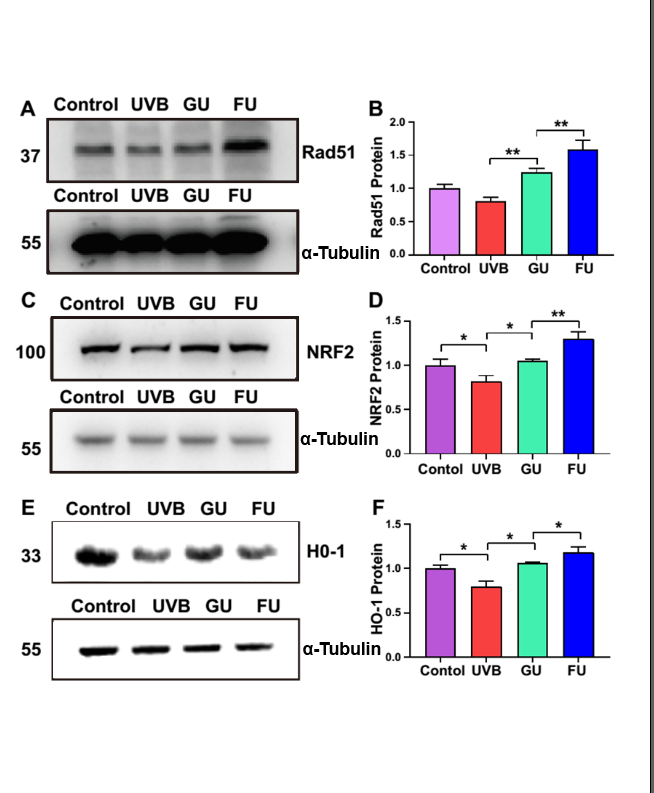


**Fig. S5.** The expression of in RAD51、NRF2 and HO-1 protein levels after UVB exposure to hCECs at the same molar concentration of fullerenol and GSH treatment. A) Representative immunoblot analysis of RAD51 in treated hCECs. B) Quantification of RAD51 protein expression. C) Representative immunoblot analysis of NRF2 in treated hCECs. D) Quantification of NRF2 protein expression. E) Representative immunoblot analysis of HO-1 in treated hCECs. F) Quantification of HO-1 protein expression. Error bars are mean ± SEM of triplicate experiments. *P < 0.1, **P < 0.01, ***P<0.001, ****P < 0.0001 using one-way ANOVA and post-hoc Tukey’s test.


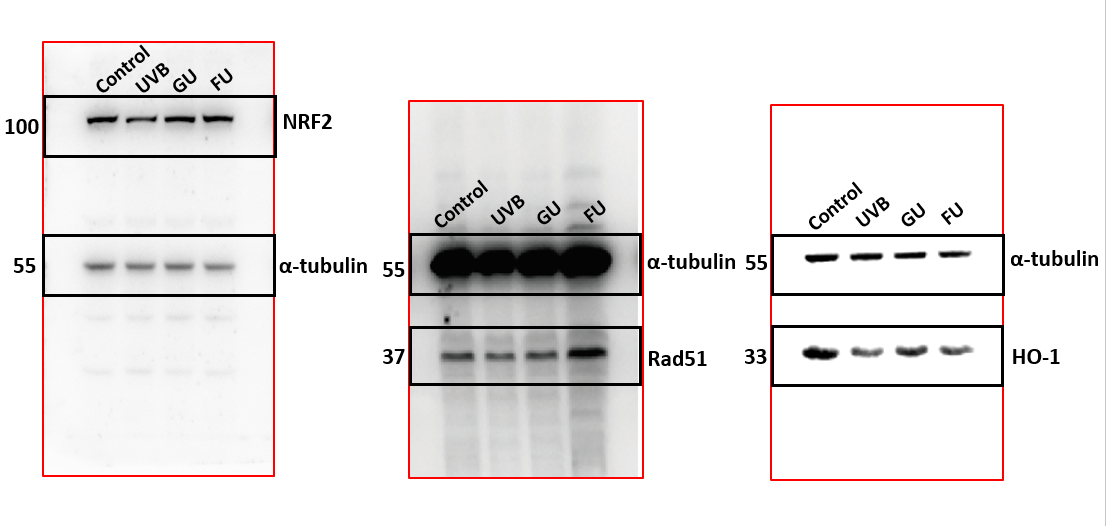


**Fig. S6. The expression of in RAD51、NRF2 and HO-1 protein levels with uncropped full-length blots after UVB exposure to hCECs at the same molar concentration of fullerenol and GSH treatment.** A) Representative immunoblot analysis of NRF2 in treated hCECs. B) Quantification of RAD51 protein expression. C) Representative immunoblot analysis of HO1 in treated hCECs.
